# Assessing Levels of Lymphedema Awareness Among Women With Breast Cancer in King Abdulaziz University Hospital, Jeddah

**DOI:** 10.7759/cureus.78046

**Published:** 2025-01-27

**Authors:** Sarah Alyamani, Reem Alghamdi, Raghad Rayes, Heba Yassin, Latifah Alhamed, Aminah Almadani, Husain Jabbad, Hattan Aljaaly

**Affiliations:** 1 Medical School, King Abdulaziz University Faculty of Medicine, Jeddah, SAU; 2 Surgery, King Abdulaziz University Faculty of Medicine, Jeddah, SAU; 3 Plastic Surgery, King Abdulaziz University Faculty of Medicine, Jeddah, SAU

**Keywords:** breast neoplasms, cultural influences, healthcare dynamics, healthcare-seeking behaviour, lymphedema awareness, postoperative complications

## Abstract

Introduction

Breast cancer poses a global health challenge, requiring a comprehensive approach beyond diagnosis and treatment. Postoperative complications, especially upper limb lymphedema, present intricate challenges for survivors, impacting physical, emotional, and daily life aspects. Our research in Jeddah, Saudi Arabia, delves into cultural and healthcare dynamics, exploring demographic influences on lymphedema.

Materials and methods

A descriptive quantitative study with a cross-sectional design was conducted among female patients with breast cancer in Saudi Arabia. It included patients who underwent unilateral breast surgery with lymph node excision. Data was analyzed using the SPSS program (IBM Corp., Armonk, NY).

Results

Our study included 76 participants. Lymphedema was diagnosed in 38.2% of participants. More than half (52.8%) of participants had some knowledge of lymphedema. For instance, 52.6% recognized the impact of hygiene, and 81.6% understood the heightened risk of arm damage. Awareness percentages were also notable for factors like tight shirt pressure (71.1%), straining the arms (86.8%), and the association of being overweight with lymphedema (55.3%). Almost half of the participants recognized the physical therapy and rehabilitation department to be responsible for lymphedema treatment. The mean awareness score was 5.34 ± 1.56. Only 9.2% achieved a good awareness level, while 59.2% had fair awareness, and 31.6% had poor awareness.

Conclusion

The prevalence of lymphedema, coupled with proactive healthcare-seeking behavior, underscores the need for targeted educational interventions. While the majority recognized the importance of treatment, awareness gaps persisted, especially regarding risk-reduction activities.

## Introduction

Management of breast cancer, a global health challenge [1], requires a comprehensive approach that extends beyond initial diagnosis and treatment. Postoperative complications, particularly upper limb lymphedema [2], represent intricate challenges that demand nuanced solutions. Upper limb lymphedema, characterized by arm swelling and discomfort [3], emerges as a significant concern for breast cancer survivors. This complication typically stems from the disruption of the lymphatic system during surgery, particularly when lymph nodes are removed or radiation therapy is administered [4]. Such interventions, while crucial in treating breast cancer, can impede the normal flow of lymphatic fluid, leading to its accumulation in the arm. Compromised lymphatic drainage results in the characteristic swelling and discomfort associated with upper limb lymphedema. The severity of this condition can vary, and its onset may not be immediate, often manifesting weeks, months, or even years after surgery [5]. Factors such as the extent of lymph node removal, the type of surgery, and the use of radiation therapy contribute to the risk of developing lymphedema [6]. Beyond its physical manifestations, lymphedema’s potential to impede daily functioning and compromise the overall quality of life underscores the urgency of unravelling its complexities [7].

The challenges posed by upper limb lymphedema extend far beyond its visible symptoms. Beyond the physical discomfort, this complication can impact the emotional and psychological well-being of breast cancer survivors [8]. Daily tasks, once taken for granted, may become daunting, and the overall quality of life may be compromised. Recognizing the multifaceted nature of these challenges [9], our research aims to provide a detailed understanding of the factors influencing lymphedema development and the subsequent nuances in its management.

In the dynamic world of breast cancer care, Jeddah, situated in the Kingdom of Saudi Arabia, stands out as a lively hub [10]. As this city becomes a central place for breast cancer treatment, it’s crucial to thoroughly understand how lymphedema develops and how to manage it. Jeddah’s special mix of cultural diversity and healthcare complexities requires a deep exploration to customize interventions effectively. Age, nationality, educational background, and marital status play roles in shaping the varied experiences of breast cancer survivors [11]. Our study, aware of this diversity, aims to uncover how these demographic factors connect with the occurrence and seriousness of upper limb lymphedema.

We sought data on the participants’ handedness, the duration since breast cancer onset, and the specific treatments undergone post-surgery, seeking to identify subtle associations that may impact the development and management of lymphedema. Beyond addressing the physical aspects of lymphedema, our goal is to contribute to the development of tailored interventions, educational programs, and support mechanisms that holistically enhance the well-being of individuals navigating the aftermath of breast cancer surgery.

## Materials and methods

Study design and setting

A descriptive quantitative cross-sectional study was conducted to evaluate the level of breast cancer-related lymphedema (BCRL) awareness among women in Saudi Arabia. The study took place in King Abdulaziz University Hospital (KAUH), Jeddah, a tertiary healthcare center providing comprehensive cancer care.

Participants and sampling

Participants were recruited over a three-month period using a convenience sampling method. Inclusion criteria consisted of women aged 18 years or older, diagnosed with breast cancer, who had undergone unilateral breast cancer surgery with lymph node excision at least six months prior to recruitment, completed all chemotherapy and radiotherapy treatments, showed no current evidence of cancer, and were able to answer the questionnaire and provide informed consent. Exclusion criteria included male patients, individuals with serious systemic illnesses (e.g., kidney failure, hepatic dysfunction), neurological or psychological impairments, abnormalities or vascular diseases in the upper extremities, bilateral breast cancer due to loss of comparison between the two limbs, and inability to consent or respond.

Recruitment process

Eligible participants were identified through hospital medical records and oncology departments. Informed consent was obtained electronically and verbally before participants completed the survey, ensuring voluntary participation. Ethical approval for the study was granted by the Biomedical Institutional Review Board of King Abdulaziz University (approval number: HA-02-J-008).

Study instrument

Data were collected through a structured electronic questionnaire designed to capture demographic information, medical history, and BCRL awareness. The questionnaire was adapted from a validated Korean study which was provided in English [12]. The questionnaire was then translated into Arabic for a better understanding. The final version consisted of 23 multiple-choice and yes/no questions divided into four main sections: demographic data (age, education level, occupation, marital status, and dominant hand), medical history (breast cancer diagnosis, treatments received, and current treatment status), BCRL education (prior education and sources of information), and BCRL awareness (knowledge of risk factors, preventive activities, and treatment options).

To ensure reliability, the questionnaire was pilot-tested with 10 participants to confirm clarity, cultural relevance, and ease of understanding. Necessary adjustments were made before full deployment.

Ethical considerations

Ethical approval was obtained from the King Abdulaziz Biomedical Institution of Review Board and the patients were informed about the study’s objectives, the confidentiality of their responses, and their right to withdraw at any time. Informed consent was collected electronically before the survey commenced. All collected data were stored securely and accessed only by the research team.

Data collection

The questionnaire was distributed electronically using Google Forms. This platform allowed for efficient data collection, automated response tracking, and anonymity. Data collection took place over a three-month period, ensuring sufficient participant engagement.

Sample size justification

The sample size of 76 participants was determined based on prior studies with similar methodologies and findings. Although convenience sampling was used, efforts were made to ensure representation across age groups, education levels, and treatment experiences.

Statistical analysis

Data analysis was performed using SPSS version 26 (IBM Corp., Armonk, NY). Descriptive statistics were used to summarize demographic data, awareness levels, and responses to individual questions. Quantitative data were expressed as frequencies, percentages, means, and standard deviations (Mean ± SD). For inferential analysis, the chi-square test (χ2) was applied to assess the association between awareness levels and demographic or clinical variables. A p-value <0.05 was considered statistically significant. Missing data were handled through pairwise deletion to maintain the integrity of the analysis.

## Results

Table 1 shows that 42.1% of the participants were 41-50 years of age, 63.2% had Saudi nationality and 73.7% were homemakers. About 36% (36.8%) had a university education and 68.4% were married. Table 2 demonstrates that 88.2% of study participants relied on their right hand the most and 61.8% had a duration of ≥3 years since the onset of breast cancer.

**Table 1:** Distribution of studied participants according to their demographic characters (n=76)

Variable		n (%)
Age	31-40	12 (15.8)
	41-50	32 (42.1)
	51-60	(26.3) 20
	61-70	12 (15.8)
Nationality	Saudi	48 (63.2)
	Non-Saudi	28 (36.8)
Occupation	Homemaker	56 (73.7)
	Employed	20 (26.3)
Education	Middle school	16 (21.1)
	Secondary school	24 (31.6)
	University	28 (36.8)
	Other	8 (10.5)
Marital status	Widow	12 (15.8)
	Married	52(68.4)
	Divorced	12 (15.8)

**Table 2:** Distribution of studied participants according to dominant hand and duration since onset of breast cancer (n=76)

Variable		n (%)
Which hand do you rely on the most?	Left hand	9 (11.8)
	Right hand	67 (88.2)
What is the length of time since the onset of breast cancer?	<1 year	9 (11.8)
	1-<2 years	2 (2.6)
	2-<3 years	18 (23.7)
	≥3 years	47 (61.8)

Of them, 82.9% had lymphadenectomy, 67.1% underwent chemotherapy after the operation, and 76.3% underwent radiotherapy. Only 3.9% were currently undergoing chemotherapy or radiation therapy. About 38% (38.2%) were diagnosed with lymphedema and the majority (81.6%) seek medical advice if they notice any redness, swelling, itching, pain, or high temperature (Table 3).

**Figure 1:**
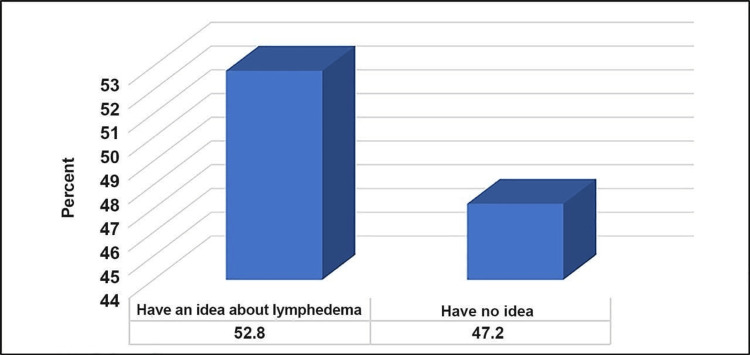
Percentage distribution of the participants knowing about lymphedema (n=76)

Figure 1 illustrates that more than half of the participants (52.8%) had some level of awareness about lymphedema. As for the participants' awareness about upper limb lymphedema, 52.6% correctly knew that lack of attention to hygiene increases the risk of infection. Of them, 81.6% knew that any damage to the arms increases the risk of injury, while 71.1% and 86.8% knew that pressure on the arm with a tight shirt and straining the arms increase the risk of injury respectively. More than half (55.3%) knew that being overweight increases the risk of lymphedema. The majority (97.4%) knew that lymphedema should be treated, while only 10.5% thought that it could not be treated (Table 4). 

**Table 3:** Distribution of the participants according to clinical history of lymphadenectomy, chemotherapy, radiotherapy, lymphedema and seeking medical when noticing upper limb alarming signs (n=76)

Variable	No; n(%)	Yes; n(%)
Have you had a lymphadenectomy?	13 (17.1)	63 (82.9)
Did you undergo chemotherapy after the operation?	25 (329)	51 (67.1)
Did you undergo radiation therapy after the operation?	18 (23.7)	58 (76.3)
Are you currently undergoing chemotherapy or radiation therapy?	73 (96.1)	3 (3.9)
Have you been diagnosed with lymphedema? (Accumulation of fluid that usually drains through the body's lymphatic system into the arms or legs)	47 (61.8)	29 (38.2)
Have you had treatment for lymphedema?	62 (81.6)	14 (18.4)
Do you seek medical advice if you notice any redness, swelling, itching, pain, or high temperature?	14 (18.4)	62 (81.6)

**Table 4:** Participants' responses to awareness items about upper limb lymphedema (n=76). N.B.: * = Correct answer

Variable	No; n(%)	Yes; n(%)
Lack of attention to hygiene increases the risk of infection.	36 (47.4)	40 (52.6) *
Any damage to the arms that may increase the risk of injury?	14 (18.4)	62 (81.6) *
Pressure on the arm with a tight shirt may increase the risk of injury.	22 (28.9)	54 (71.1) *
Straining the arms may increase the risk of injury.	10 (13.2)	66 (86.8) *
Does being overweight increase the risk of lymphedema?	34 (44.7)	42 (55.3) *
Do you think lymphedema should be treated?	2 (2.6)	74 (97.4) *
Do you think that lymphedema cannot be treated?	68 (98.5)	8 (10.5) *

**Figure 2:**
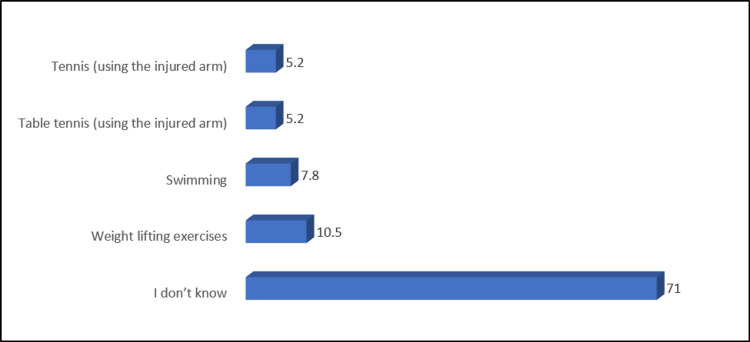
Participants' knowledge about activities helping to reduce the risk of lymphedema

**Figure 3:**
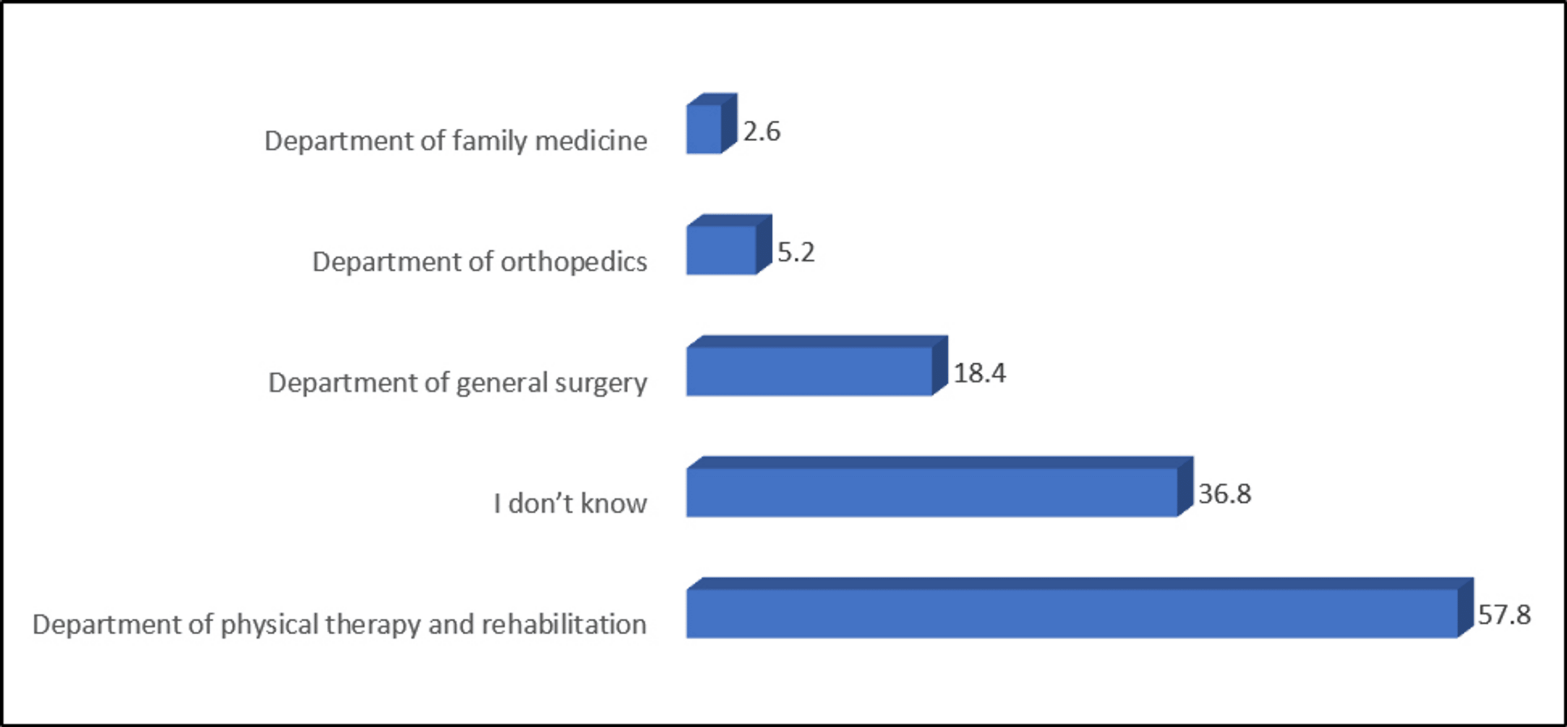
Participants' knowledge about departments or clinics that treat lymphedema

Figure 2 shows that most of the participants did not know the activities that help to reduce the risk of lymphedema. Only 7.8% mentioned swimming as the most common activity. Almost half of the participants identified the physical therapy and rehabilitation department as responsible for the treatment of lymphedema (Figure 3). 

**Figure 4:**
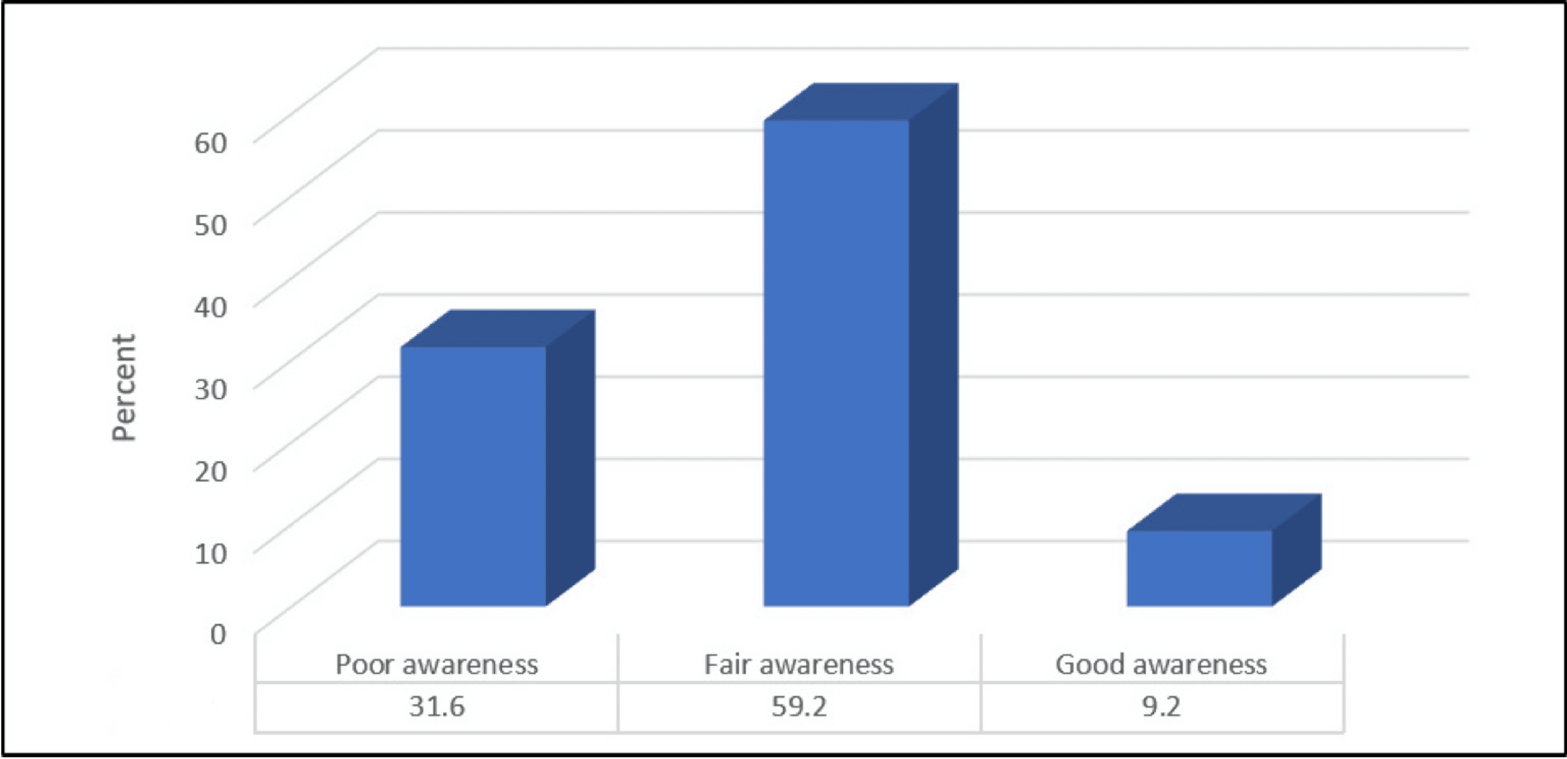
Percentage distribution of the level of awareness about upper limb lymphedema

The mean awareness score was 5.34 ± 1.56. Figure 4 shows that only 9.2% of the participants had a good awareness level about upper limb lymphedema, while 59.2% had fair awareness and 31.6% had poor awareness.

**Figure 5:**
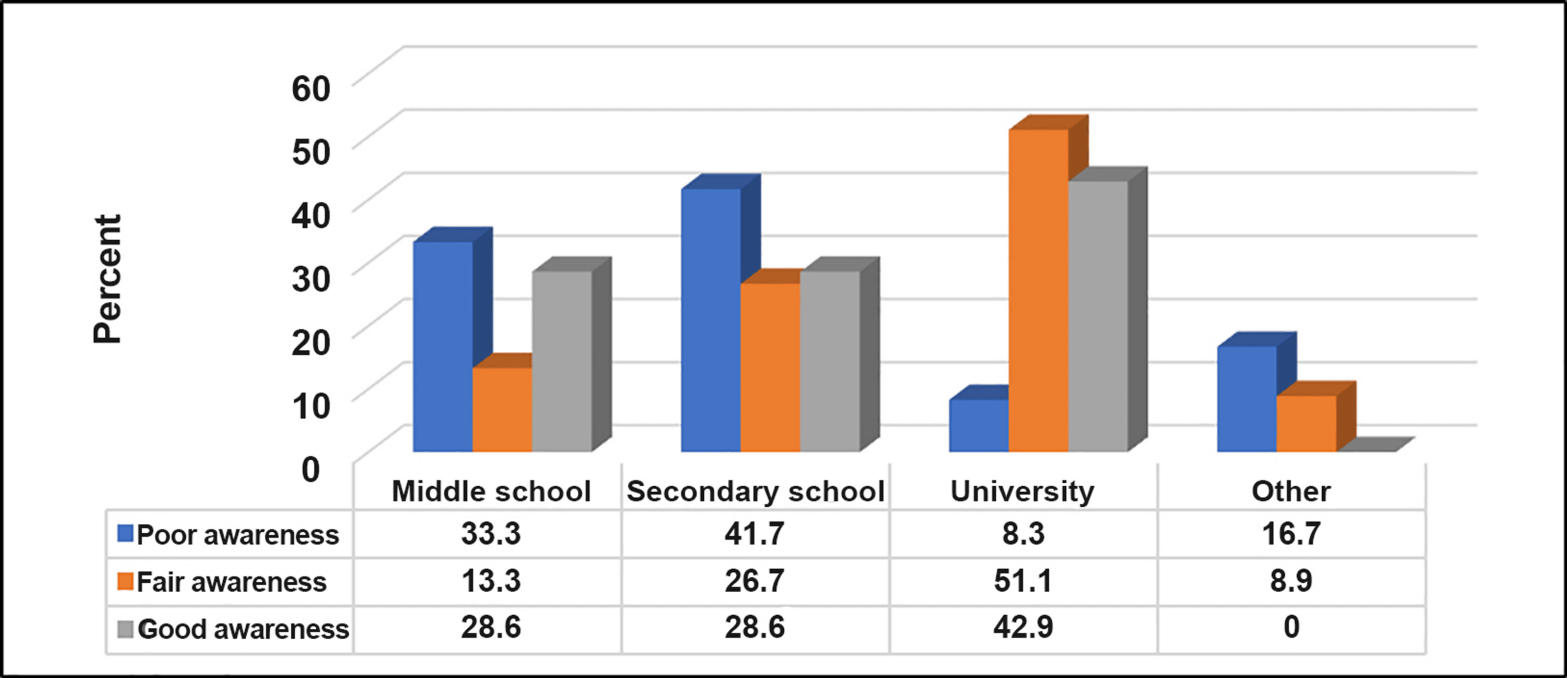
Relationship between awareness level about upper limb lymphedema and participants' educational level (n=76). N.B.: (χ2 = 13.87, p-value = 0.031)

Table 5 and Figure 5 show that a good level of awareness about upper limb lymphedema was significantly higher among participants with a university level of education (p≤0.05). On the other hand, a non-significant relationship was found between awareness level and other participants' demographics (p≥0.05).

**Table 5:** Relationship between awareness level and participants' demographics (n=76)

Variable		Awareness level			χ2	p-value
		Poor; n (%)	Fair; n (%)	Good; n (%)		
Age	31-40	4 (16.7)	6 (13.3)	2 (28.6)	6.75	0.344
	41-50	6 (25)	23 (51.1)	3 (42.9)		
	51-60	8 (33.3)	10 (22.2)	2 (28.6)		
	61-70	6 (25)	6 (13.3)	0 (0.0)		
Nationality	Saudi	14 (58.3)	31 (68.9)	3 (42.9)	2.11	0.347
	Non-Saudi	10 (41.7)	15 (31.1)	4 (57.1)		
Occupation	Housewife	20 (83.3)	32 (71.1)	4 (57.1)	2.29	0.318
	Employed	4 (16.7)	13 (28.9)	3 (42.9)		
Education	Middle school	9 (33.3)	6 (13.3)	2 (28.6)	13.87	0.031
	Secondary school	10 (41.7)	12 (26.7)	2 (28.6)		
	University	2 (8.3)	23 (51.1)	3 (42.9)		
	Other	4 (16.7)	4 (8.9)	0 (0.0)		
Marital status	Widow	4 (16.7)	6 (13.3)	2 (28.6)	5.88	0.208
	Married	14 (58.3)	35 (77.8)	3 (42.9)		
	Divorced	6 (25)	4 (6.9)	2 (28.6)		

**Figure 6:**
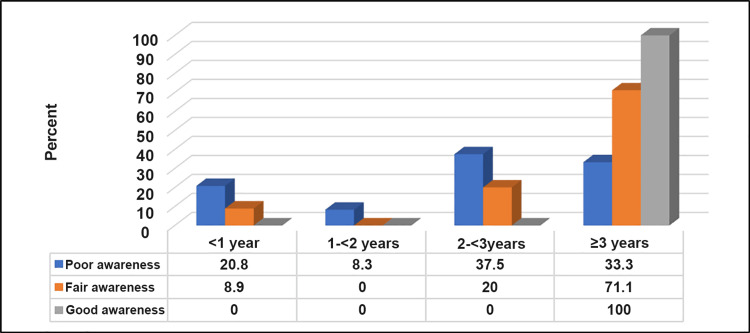
Relationship between awareness level about upper limb lymphedema and duration since breast cancer onset (n=76) N.B.: (χ2 = 16.41, p-value = 0.012)

**Figure 7:**
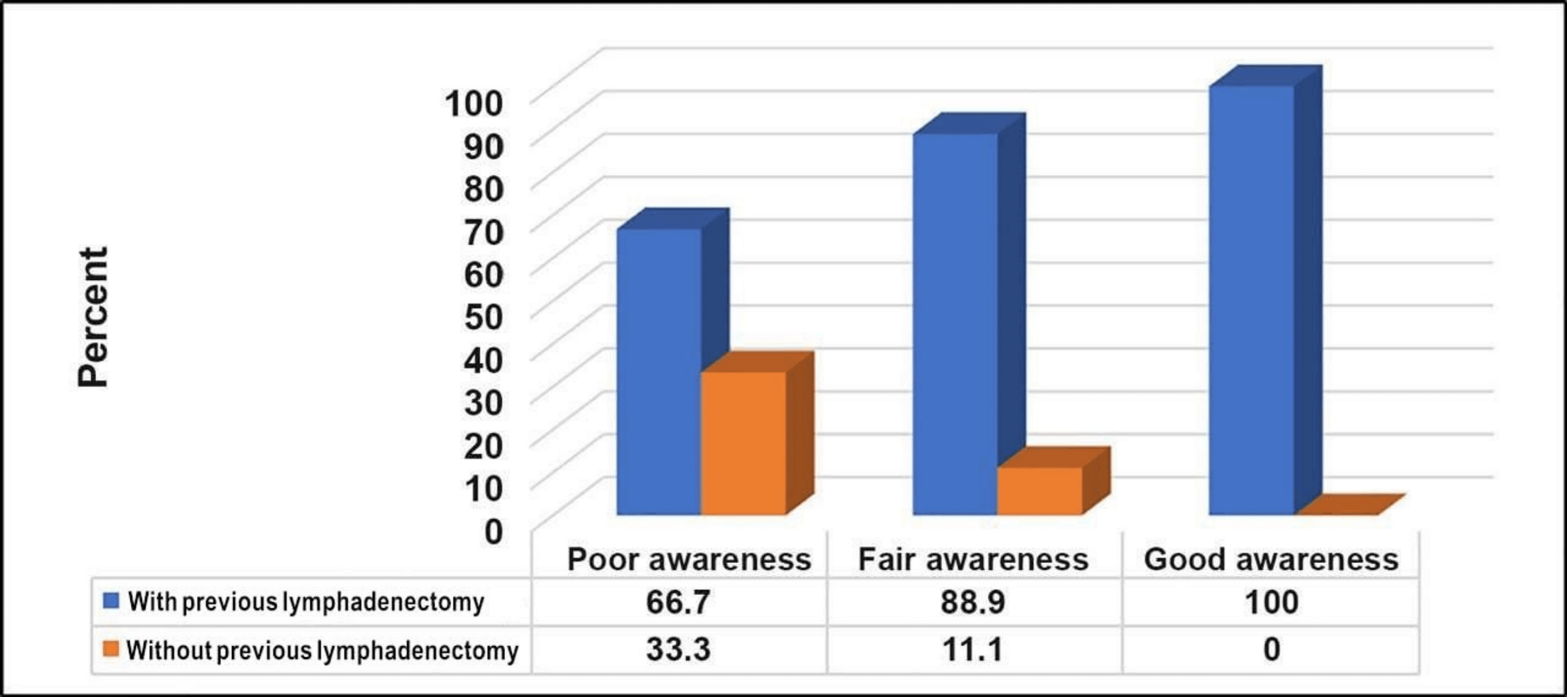
Relationship between awareness level about upper arm lymphedema and previous lymphadenectomy (n=76). N.B.: (χ2 = 7.04, p-value = 0.03)

Table 6, Figure 6, and Figure 7 show that a good level of awareness about upper limb lymphedema was significantly higher among participants who experienced breast cancer onset for ≥3 years, those who had lymphadenectomy, and those who underwent chemotherapy or radiation therapy after the operation (p=<0.05). 

**Table 6:** Relationship between awareness level and dominant hand and duration since onset of breast cancer, clinical history of lymphadenectomy, chemotherapy, radiotherapy, lymphedema and seeking medical when noticing upper limb alarming signs (n=76)

Variable		Awareness level			χ2	p-value
		Poor; n (%)	Fair; n (%)	Good; n (%)		
Which hand do you rely on the most?	Left hand	2 (8.3)	6 (13.3)	1 (14.3)	0.41	0.811
	Right hand	22 (91.7)	39 (86.7)	6 (85.7)		
The length of time since the onset of breast cancer?	<1 year	5 (20.8)	4 (8.9)	0 (0.0) 7	16.41	0.012
	1-<2 years	2 (8.3)	0 (0.0)	0 (0.0)		
	2-<3 years	9 (37.5)	9 (20)	0 (0.0)		
	≥3 years	8 (33.3)	32 (71.1)	-100		
Have you had a lymphadenectomy?	No	8 (33.3)	5 (11.1)	0 (0.0)	7.04	0.03
	Yes	16 (66.7)	40 (88.9)	7 (100)		
Did you undergo chemotherapy after the operation?	No	15 (62.5)	10 (22.2)	0 (0.0)	15.28	<0.001
	Yes	9 (37.5)	35 (77.8)	7 (100)		
Did you undergo radiation therapy after the operation?	No	11 (45.8)	7 (15.6)	0 (0.0)	10.33	0.006
	Yes	13 (54.2)	38 (84.8)	7 (100)		
Are you currently undergoing chemotherapy or radiation therapy?	No	24 (100)	42 (93.3)	7 (100)	2.15	0.341
	Yes	0 (0.0)	3 (6.7)	0 (0.0)		
Have you been diagnosed with lymphedema? (accumulation of fluid that usually drains through the body's lymphatic system into the arms or legs)	No	17 (70.8)	27 (60)	3 (42.9)	1.95	0.376
	Yes	7 (29.2)	18 (40)	4 (57.1)		
Have you had treatment for lymphedema?	No Yes	22 (91.7)	34 (75.6)	6 (85.7)	2.79	0.248
		2 (8.3)	11 (24.4)	1 (14.3)		
Do you seek medical advice if you notice any redness, swelling, itching, pain, or high temperature?	No	6 (25)	6 (13.3)	2 (28.6)	1.94	0.378
	Yes	18 (75)	39 (86.7)	5 (71.4)		

## Discussion

Our study aimed to assess the level of awareness and understanding among post-breast cancer surgery patients regarding the management of complications, with a specific focus on lymphedema. Approximately 38.2% of participants were diagnosed with lymphedema. This underscores the significant prevalence of this complication post-breast cancer surgery, consistent with the study conducted in 2018 [13]. Encouragingly, a substantial majority of those with lymphedema proactively sought medical advice upon noticing symptoms like redness, swelling, itching, pain, or elevated temperature [14]. This suggests a positive trend in prompt healthcare-seeking behavior [15].

More than half of the participants showed awareness of lymphedema, indicating a reasonable level of general knowledge within the studied population. Regarding upper limb lymphedema, participants displayed varying levels of awareness [16]. The observation that 52.6% of participants correctly recognized the role of hygiene in infection prevention shows a moderate level of awareness of this crucial aspect [17]. The heightened awareness regarding the increased risk of injury with any arm damage reflects a commendable understanding of the potential consequences associated with such incidents.

Furthermore, the substantial knowledge demonstrated by participants about specific risk factors, including the impact of tight shirt pressure and straining the arms, suggests a nuanced understanding of potential triggers for lymphedema [18]. The recognition of the association between being overweight and an elevated risk of lymphedema by over half of the participants is noteworthy, emphasizing the importance of weight management in lymphedema prevention [19].

However, the study also highlights areas for improvement in overall awareness, indicating that there are specific aspects of upper limb lymphedema that may require targeted education. These findings underscore the significance of tailored interventions to enhance general awareness and address specific knowledge gaps identified in the study, ultimately contributing to better preventive practices and management of upper limb lymphedema within the studied population [20].

The overwhelming majority recognized that lymphedema should be treated, reflecting a positive attitude toward addressing this condition. Around 10.5% believed that lymphedema cannot be treated. This misconception presents an opportunity for targeted education to improve understanding and dispel myths surrounding the manageability of lymphedema [21, 22].

The study indicates a need for more awareness among participants regarding activities that help reduce the risk of lymphedema. This highlights a crucial area for targeted education, as informed lifestyle choices can play a significant role in mitigating the risk of this post-operative complication [23]. Approximately half of the participants correctly identified the physical therapy and rehabilitation department to be responsible for treating lymphedema [24]. This suggests a moderate level of awareness about the appropriate healthcare resources for managing this condition.

The mean awareness score indicates a moderate overall level of awareness within the study population. While there is room for improvement, it suggests a baseline awareness that can be built upon through targeted educational interventions. Only a small proportion of participants demonstrated a good awareness level about upper limb lymphedema. On the positive side, the majority had a fair awareness level. However, it is concerning that a significant portion had poor awareness. This underscores the need for focused educational efforts to enhance understanding, particularly among individuals with lower awareness scores [25].

A significant association was found between a good level of awareness about upper limb lymphedema and participants with a university-level education. This underscores the role of higher education in fostering awareness and suggests that educational interventions may benefit from tailored approaches for individuals with varying educational backgrounds [18, 26].

A longer duration since breast cancer onset was significantly correlated with a higher awareness level. This suggests that over time, individuals may gain a deeper understanding of post-operative complications and the importance of lymphedema awareness [27]. Participants who underwent lymphadenectomy and received chemotherapy or radiation therapy after the operation also demonstrated a significantly higher level of awareness. This could be attributed to increased exposure to healthcare information and heightened awareness due to the nature of their treatment experiences [28].

Certain limitations should be acknowledged. The cross-sectional nature of our research design restricts our ability to establish causal relationships between demographic factors and awareness levels. The reliance on self-reported data introduces the potential for recall bias, as participants may not accurately recall specific details of their postoperative experiences. Additionally, the study’s single-center focus may limit the generalizability of findings to a broader population. The survey’s reliance on closed-ended questions may have constrained the depth of responses, potentially overlooking nuanced perspectives that could influence postoperative care.

## Conclusions

The prevalence of lymphedema, coupled with proactive healthcare-seeking behaviour, underscores the need for targeted educational interventions. While the majority recognized the importance of treatment, awareness gaps persisted, especially regarding risk-reduction activities. This highlights the influence of education and treatment history on awareness levels. These findings emphasize the importance of tailored educational programs to enhance overall awareness, facilitate early intervention, and optimize postoperative outcomes for breast cancer patients.
